# Changes in the Prevalence of Metabolic Syndrome and Its Components as Well as in Relevant Preventive Medication between 2006 and 2018 in the Northeast Hungarian Population

**DOI:** 10.3390/jpm11010052

**Published:** 2021-01-16

**Authors:** Peter Piko, Judit Dioszegi, Janos Sandor, Roza Adany

**Affiliations:** 1 MTA-DE Public Health Research Group, University of Debrecen, 4032 Debrecen, Hungary; piko.peter@med.unideb.hu (P.P.); dioszegi.judit@med.unideb.hu (J.D.); 2 Department of Public Health and Epidemiology, Faculty of Medicine, University of Debrecen, 4032 Debrecen, Hungary; sandor.janos@med.unideb.hu

**Keywords:** metabolic syndrome, prevalence, Hungarian, diabetes, hypertension, preventive medication, targeted public health strategy

## Abstract

Metabolic syndrome (MetS) is a worldwide problem with severe health consequences. In this study, we examine the changes in the prevalence of MetS and its components in two disadvantaged counties of Northeastern Hungary. Two health examination surveys were performed in the Hungarian population aged 20–64 years in 2006 (*n* = 450) and 2018 (*n* = 397) and the data were compared to each other. It was found that the prevalence of MetS increased significantly in the period examined (from 34.9% to 42.2%, *p* = 0.035) due to the increased prevalence of raised blood pressure (from 45.6% to 57.0%, *p* = 0.002) and raised fasting glucose concentration (13.2% vs. 24.8%, *p* < 0.001). The increase mainly affects the younger (20–34 years old) age group (12.1% in 2006 vs. 31.6% in 2018, *p* = 0.001). It is quite alarming that the prevalence of MetS and its components has increased significantly in the last decade, while the prevalence of preventive medication is unchanged (antihypertensive and antidiabetic treatments) or even significantly decreased (lipid-lowering medication). Consequently, the number of individuals untreated for hypertension and metabolic disturbances is severely increased. A targeted public health strategy is desperately needed to prevent further worsening the situation.

## 1. Introduction

Noncommunicable diseases (NCDs) are emerging as a health concern worldwide. For NCDs, the most robust risk indicator is the metabolic syndrome (MetS) as a consequence of complex disturbances in lipid and carbohydrate metabolic pathways in connection with insulin resistance and certain inflammatory processes [1] and, presently, they can be considered as a worldwide pandemic [2,3]. MetS is defined by a cluster of interconnected factors which directly and indirectly increase the risk of cardiovascular diseases (CVDs), cancer, nonalcoholic fatty liver disease, dementia, infertility, type 2 diabetes mellitus (T2DM) and other diseases [4]. As is defined by the International Diabetes Federation, MetS occurs when a person has abdominal obesity and at least two of the following measurements: elevated triglyceride (TG) levels or treated lipid disorder, reduced high-density lipoprotein cholesterol (HDL-C) levels or treated lipid disorder, raised blood pressure (BP) or treated hypertension, and elevated fasting plasma glucose (FPG) concentration or previously diagnosed diabetes mellitus [5]. In addition to genetic factors, the excessive intake of energy-dense foods and drinks combined with reduced physical activity are to be blamed for the increasing prevalence of obesity, the core component of MetS [6,7].

Currently, Hungary is the fourth most obese country worldwide (behind the United States, Mexico, and New Zealand), and it is facing the most serious obesity problem in the European Union [8]. One in five adults was obese in 2017, and the situation is further aggravated by the very high rate of obesity not only among the adult population but also among children and adolescents [9]. Seventy percent of adults reported not consuming any vegetables on a daily basis, and 60% indicated they did not eat fruit daily in Hungary [10], and although they lead physically active lives, the dominant forms of their physical activity are linked to work and housework [11].

Previous studies have shown that obesity [12,13] and T2DM [14] are mainly determined by the environment and not by genetic factors, while there are clearly genetic reasons behind the prevalence of elevated blood pressure [15], low HDL-C levels [16,17,18] and age of onset for T2DM [19] in the Hungarian general population.

Many people live with the high risk of or with metabolic syndrome in Hungary, which promotes the higher risk for the development of CVDs, T2DM, and other diseases [20]. Half of all deaths in Hungary are caused by behavioral risk factors such as alcohol consumption, poor diet, low leisure time physical activity, and smoking. The standardized death rate for CVDs—the leading cause of death in the country—was 588.15 per 100,000 inhabitants in Hungary in 2017 (the fifth worst among the EU-28 countries), which is 1.6 times higher than the EU average (369.46 deaths per 100,000) [21]. The standardized prevalence rate of type 2 diabetes increased from 4.2% to 6.4% in the Hungarian adult population in the period between 2001 and 2014 [22], and the number of deaths from T2DM has continuously been increasing [23].

Our research group conducted a survey in 2006 based on which the frequency of metabolic syndrome and its components in the general Hungarian population was established [24,25]. In 2018, we conducted a complex health survey in two counties in Northeastern Hungary (Hajdú-Bihar (HB) and Szabolcs-Szatmár-Bereg (SSB)) and created a database of half a million records [26]. With the help of these two databases, it was possible to perform a comparative analysis.

The aims of our present study are: (1) to examine how the prevalence of metabolic syndrome and its components changed between 2006 and 2018; (2) to identify the components, as well as the sex and age groups most affected by these changes; (3) to examine how the rate of drug treatments associated with metabolic syndrome has changed, and (4) to suggest targeted preventive interventions based on our findings.

## 2. Materials and Methods

### 2.1. Study Populations for Comparison from 2006 and 2018

In the survey were conducted in 2018, individuals aged 20–64 years were randomly drawn by general practitioners involved in the General Practitioners’ Morbidity Sentinel Stations Programme (GPMSSP), a population-based disease monitoring system [27] in two counties of Northeast Hungary (HB and SSB). Twenty-five random individuals from 20 randomly selected general practitioners’ practices were invited to participate in the study. Other individuals were enrolled from the same household for subjects who could not be reached, but drawing another person was not allowed if someone refused to participate. For more details on the study population, see our previous article [26].

Based on the GPMSSP, a questionnaire and a physical examination-based survey was carried out in 2006 [24,27], which provided the reference population for 2006 to our present study. Participants, 20–64 years of age, were drawn from the people of counties representing the whole Hungarian general population by geographic, sex and age distributions. A more detailed explanation of the sampling applied and the survey data collected are described in the Hungarian Metabolic Syndrome Survey [24].

As a part of both surveys, physical examinations were carried out, and information was collected on each participant’s medical history and socio-demographic characteristics. Blood samples were taken and laboratory tests relevant to metabolic syndrome were performed.

Studies have demonstrated that geographical location and socioeconomic conditions could determine the frequency of MetS and its components [28,29,30,31]. In order to eliminate the effects of the different geographical features of the country, individuals from HB and SSB counties were selected for further analysis from the 2006 sample population.

**Figure 1:**
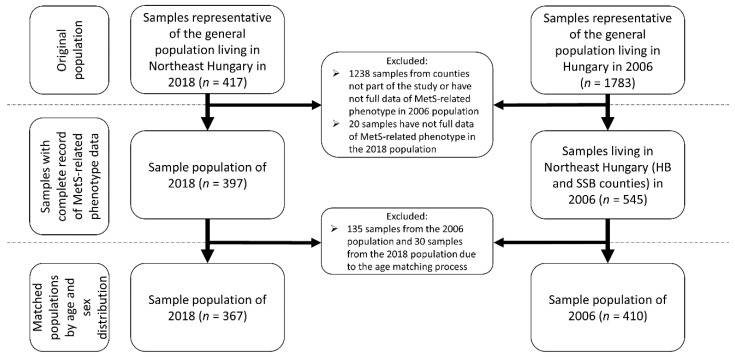
Process of sample selection of the study populations.

For the study populations, only those samples were selected that had a complete record of MetS-related phenotype data. These data include age, sex, waist circumference, fasting glucose level, triglyceride (TG) level, high-density lipoprotein cholesterol (HDL-C) level, present antihypertensive, antidiabetic and/or lipid-lowering treatments. To avoid the biasing effect of biological differences due to age and sex, the sample populations were matched according to age and sex distributions. For more details on sample selection, see Figure 1.

### 2.2. Determination of the Prevalence of Metabolic Syndrome and Its Components

The prevalence rates of MetS and its components were defined by accepting the International Diabetes Federation (IDF) definition [32]. According to this definition, somebody is considered to have metabolic syndrome if he/she has central obesity (waist circumference: ≥94 cm for men and ≥80 cm for women—for Europid population) as sine qua non combined with two or more of the following four factors:

1. raised concentration of triglycerides (≥1.7 mmol/L) or specific treatment for this lipid abnormality;
2. reduced concentration of HDL cholesterol: (<1.03 mmol/L in men and <1.29 mmol/L in women) or specific treatment for this lipid abnormality;
3. raised blood pressure (systolic blood pressure ≥ 130 mmHg and/or diastolic blood pressure ≥ 85 mmHg) or treatment of previously diagnosed hypertension;
4. raised fasting plasma glucose concentration (≥5.6 mmol/L) or previously diagnosed type 2 diabetes.

### 2.3. Statistical Analyses

All statistical tests were conducted by using IBM SPSS (version 26, IBM Company, Armonk, NY, USA) software. Prevalence data (sex, age group, treatments/therapies, MetS, and its components) for the two samples were compared by an χ^2^ test. Mann–Whitney U tests were used to assess the statistical difference of variables (biochemical and physical parameters) among the groups. Subjects were categorized by age as follows: 20–34, 35–49, and 50–64 years. Differences in prevalence were evaluated by the 95% CI presented. Multivariate logistic regression analyses (adjusted for age and sex) were used to examine the risk for MetS and its components between the sample populations from 2006 and 2018. Generally, the conventional *p* threshold of 0.05 was used.

### 2.4. Ethical Statement

All subjects had given their informed consent for inclusion before they participated in the study. The study was conducted in accordance with the Declaration of Helsinki, and the protocol was approved by the Ethics Committee of the Hungarian Scientific Council on Health (Reference No.: 2462–2006 for study population from 2006 and 61327-2017/EKU for study population from 2018).

## 3. Results

### 3.1. Characteristics of Study Populations for Comparative Analysis Used to Estimate the Prevalence of Metabolic Syndrome

#### 3.1.1. Anthropometric and Demographic Characteristics of the Study Populations

Samples from the two counties used for this study (HB and SSB) as reference were selected from the 2006 Hungarian general population. Following the selection process, the two study populations (from 2006 and 2018) were matched by age and sex. For more details on anthropometric data of study populations, see Table S1.

#### 3.1.2. Parameters used to Estimate the Prevalence of Metabolic Syndrome in the Study Populations

A significant difference was measurable for average plasma fasting glucose levels (4.6 mmol/L in 2006 vs. 5.2 mmol/L in 2018, *p* < 0.001) between the 2006 and 2018 sample populations. The prevalence of lipid-lowering therapy also differed significantly between the two study groups (15.9% in 2006 vs. 7.1% in 2018, *p* < 0.001). These significant changes can be observed for both sexes. The characteristics of biochemical and physical parameters of study populations can be seen in more detail in Table 1 and in Table S2A,B by sex.

### 3.2. The Prevalence of MetS and Its Components in the Study Populations

The prevalence of MetS increased significantly between 2006 and 2018 (from 34.9% to 42.2%, *p* = 0.035). Central obesity became more frequent in this period, but the extent of this was not significant (from 70.7% to 75.5%, *p* = 0.137). There was no significant change in lipid parameters including fasting TG, HDL-C, total cholesterol (in 2006: 5.1 mmol/L, in 2018: 5.0 mmol/L; *p* = 0.233) either. Significant changes in the frequency of raised blood pressure or treated hypertension (from 45.6% to 57.0%, *p* = 0.002) and raised fasting plasma glucose concentration or previously diagnosed diabetes mellitus (from 13.2% to 24.8%, *p* < 0.001) were observed.

In males, the prevalence of MetS increased between 2006 and 2018, although not significantly (from 36.7% to 40.8%, *p* = 0.451). The prevalence of central obesity raised the concentration of triglycerides (or treated for lipid abnormality) and the reduced HDL cholesterol level (or treated for lipid abnormality) did not change significantly during the examined time period. The prevalence of raised blood pressure (or the treatment of previously diagnosed hypertension) increased at a level close to significant (*p* = 0.051), from 49.7% to 60.6%. The prevalence of raised fasting plasma glucose concentration (previously diagnosed type 2 diabetes) was significantly—1.57 times—higher in 2018 than in 2006 (increased from 17.0% to 26.5%, *p* = 0.036).

In females, the prevalence of MetS increased significantly between 2006 and 2018 (from 33.5% to 43.2%, *p* = 0.034) and an increase could be observed for all parameters of MetS. The prevalence of central obesity increased from 76.8% to 82.7% (*p* = 0.119), the prevalence of raised triglyceride level or treated lipid disorder from 33.1% to 35.0% (*p* = 0.661), and the prevalence of reduced HDL cholesterol level or treated lipid disorder increased from 38.6% to 39.6% (*p* = 0.841) between 2006 and 2018, which is not statistically significant. The prevalence of raised blood pressure or treated hypertension and that of raised fasting plasma glucose concentration or previously diagnosed diabetes mellitus increased significantly (from 42.5% to 54.6%, *p* = 0.010 and from 10.3% to 23.6%, *p* < 0.001, respectively) in the period examined. For more details, see Table 2.

It should be highlighted that while both the prevalence of raised blood pressure and that of raised fasting plasma glucose concentration was significantly increased (*p* = 0.002 and *p* < 0.001, respectively) in the period examined (see Table 2), no change was observed neither in the prevalence of antihypertensive nor in that of antidiabetic treatment (see Table 1B and Table S2B). More strikingly, as we also demonstrate in these tables, although the prevalence of lipid disturbances remained as high for 2018 as they were in 2006, the prevalence of lipid-lowering medication decreased significantly (15.9% vs. 7.1%, *p* < 0.001), they was reduced by more than half.

### 3.3. Age Specific Prevalence of Metabolic Syndrome and Its Components in the Study Populations

An increase in the prevalence of metabolic syndrome was observed in all three age groups (20–34, 35–49, and 50–64 years) between 2006 and 2018, but it was statistically significant only in the 20–34-year-old group (from 12.1% to 31.6%, *p* = 0.001). With the prevalence of raised triglyceride levels or treated lipid disorders being the only exception, a significant increase was observed in all components of MetS in this age group. The prevalence of central obesity increased from 44.4% to 62.2% (*p* = 0.012), raised blood pressure or treated hypertension increased from 13.1% to 36.7% (*p* < 0.001), raised fasting plasma glucose concentration or previously diagnosed diabetes mellitus increased from 1.0% to 7.1% (*p* = 0.029), while the prevalence of reduced HDL cholesterol level or treated lipid disorder increased from 21.2% to 30.6% (*p* = 0.016) in the examined period.

In the 35–49-year-old age group, a significant difference was measured between the 2006 and 2018 study populations in the prevalence of raised blood pressure or treated hypertension, from 36.6% to 50.4% (*p* = 0.023), and that of raised fasting plasma glucose concentration or previously diagnosed diabetes mellitus increased from 6.3% to 20.8% (*p* < 0.001).

For the 50–64-year-old age group, a significant difference could be observed only in the prevalence of raised fasting plasma glucose concentration or previously diagnosed diabetes mellitus (from 26.0% to 40.3, *p* = 0.007). For more details, see Table 3.

### 3.4. Results Related to Risk for Development of MetS and Its Components Obtained in Multivariate Regression Models

Logistic regression analysis was used to estimate the risk for the development of MetS and its components in each age group in the 2018 population compared to the 2006 population. The risk of metabolic syndrome (odds ratio (OR) = 1.21, *p* = 0.001), central obesity (OR = 1.12, *p* = 0.008), and reduced HDL-C levels or treated lipid disorder (OR = 1.12, *p* = 0.012) was significantly higher among the 20–34-year-old age group of 2018 compared to that of 2006. The risk of raised BP or treated hypertension was significantly higher in the age groups of 20–34- (OR = 1.24, *p* < 0.001) and 35–49-year-olds (OR = 1.08, *p* = 0.047) in the 2018 sample population. The change in the prevalence rates of elevated FPG concentration or previously diagnosed diabetes mellitus in all three age groups indicates at least a closely significant higher risk in the 2018 population compared to the 2006 study group (20–34 years: OR = 1.33, *p* = 0.066; 35–49 years: OR = 1.21, *p* = 0.001; 50–64 years: OR = 1.10, *p* = 0.009). For more details, see Table 4.

### 3.5. The Change of the Proportion of Those with Untreated Metabolic Syndrome Component in Sample Populations from 2006 and 2018

We examined how the prevalence of untreated individuals for the four treatable components of MetS had changed in study populations by sex between 2006 and 2018.

The prevalence of untreated individuals with raised blood pressure (from 34.8% in 2006 to 47.9% in 2018, *p* = 0.008), raised triglyceride (from 60.6% in 2006 to 82.1% in 2018, *p* < 0.001) and reduced high-density lipoprotein cholesterol levels (from 59.1% in 2006 to 81.2% in 2018, *p* < 0.001) was significantly higher in the sample population of 2018 compared to that of 2006. As opposed to this, there was no significant change in the prevalence of untreated individuals with raised fasting plasma glucose between 2006 and 2018 (from 64.8% to 74.7%, *p* = 0.203).

Among women, the prevalence of untreated individuals was significantly higher for all four components in the 2018 sample population compared to 2006. In men, a significant increase was only measurable for the two lipid parameters (elevated TG and reduced HDL-C levels) in samples from 2018. For more details, see Table 5A,B.

## 4. Discussion

This study reports the change in the prevalence of metabolic syndrome and its components among adults (20–64 years) living in two counties of Northeastern Hungary between 2006 and 2018.

The prevalence of MetS was significantly higher in the Northeastern Hungarian population in 2018 compared to data from 2006 (34.9 % vs. 42.2%, *p* = 0.035). This is due to the significantly increased prevalence of raised blood pressure or treated hypertension (from 45.6% to 57.0%, *p* = 0.002) and raised fasting plasma glucose concentration or previously diagnosed diabetes mellitus (from 13.2% to 24.8%, *p* < 0.001), but the fact that the prevalence of abdominal obesity also increased (from 70.7% to 75.5%, *p* = 0.137) cannot be neglected either.

Having examined the change in the prevalence of MetS by sex, there was no significant increase among males (from 36.7% to 40.8%, *p* = 0.451), while the prevalence was increased by nearly 10% in females (from 33.5% to 43.2%, *p* = 0.034) between 2006 and 2018. In both sexes, this phenomenon can be explained by the increased prevalence of raised BP or treated hypertension (in males from 49.7% to 60.6%, *p* = 0.051; in females from 42.5% to 54.6%, *p* = 0.010) and raised FPG concentration or previously diagnosed diabetes mellitus (in males from 17.0% to 26.5%, *p* = 0.036; in females from 10.3% to 23.6%, *p* < 0.001).

Adverse change in the frequency of metabolic syndrome is consistent with literature data. Three studies [7,33,34] examined changes in MetS frequency in the U.S. population between 1988 and 2016. In this period, the prevalence of MetS increased from 23.1% to 36.9%. One study examined changes in the frequency of MetS in Jordan between 2009 and 2017. Similar to the U.S. studies, there was an increase in the prevalence of MetS from 38% to 44% over the study period [35]. In Korea, there was an increasing trend in the prevalence of metabolic syndrome between 1998 and 2007 (from 24.9% to 31.3%) [36]. This trend stopped between 2008 and 2013 as a result of measures taken by the Korean government and the Korean National Assembly (disease prevention and approved laws on health promotion) [37].

Age-specific examination of metabolic syndrome and its components showed a remarkably unfavorable picture in the 20–34-year-old age group from 2018 compared to 2006. Comparison between the prevalence of MetS and its components in the same age group clearly shows that risk for MetS was 1.21 times (*p* = 0.001) higher among those aged 20–34 in 2018 than in 2006. No significant change in the prevalence of MetS was measurable in the other two age groups. Among the 20–34-year-old subjects, there was a marked increase in the prevalence of obesity, high blood pressure, elevated blood glucose, and decreased HDL-C levels.

The results we describe are in harmony with what Nolan and colleagues observed in their study [38] where the MetS prevalence measured among young adults averaged 7% (between 0.7% and 22%) based on IDF criteria. They also highlight that decreased HDL-C levels can be considered as an important MetS predictive marker among young adults. In our present study, the prevalence of decreased HDL-C levels also increased significantly, from 21.21% to 36.73% (*p* = 0.016), in the subgroups aged 20–34 years, while there was no significant change in the other age groups between 2006 and 2018. High prevalence of MetS was also detected among young adults (aged 18–30 years) in a study recently published [39] on the Ellisras (town in the Limpopo province of South Africa) rural population, and increasing prevalence of MetS even among children and adolescents seems to be a worldwide phenomenon [40,41,42]. These observations are interpreted as outcomes of severe nutritional changes and missing preventive interventions targeting healthy diet [40,41,42]. Several studies have found that young adults are at increased risk for the development of cardiovascular diseases [43,44,45] and for mental disorders [46,47] in the presence of metabolic syndrome.

The situation is further aggravated by the fact that although preventive medication (statin therapy, antihypertensive treatments, and glucose-lowering therapy) is available to prevent or at least mitigate the development of MetS components, the proportion of untreated at-risk individuals is increased in 2018 compared to the 2006 sample. Half of those with high blood pressure and two-thirds of those with elevated fasting glucose and TG levels or reduced HDL-C levels remained untreated in the 2018 sample population. Our previous results show that preventive medication by statin [48], as well as antihypertensive treatments with new generation drugs [49] and antithrombotic preventive medication [50], are insufficient especially in the case of individuals living in socio-economically deprived areas such as the counties involved in the present study [51].

These results clearly show that there is a large increase in the risk and thus the prevalence of metabolic syndrome and its components in the general population living in Northeast Hungary, especially among young adults (20–34 years old).

Metabolic syndrome places a significant burden on the health care system and the associated health problems, such as illnesses and shortened lifespans, also impose a financial burden on Hungary and the whole EU. This may become even worse due to the current pandemic because the metabolic syndrome is a risk factor influencing the progression and prognosis of Coronavirus disease 2019 (caused by the Severe Acute Respiratory Syndrome—Coronavirus-2) [52].

Considering these results, it is essential to develop a nationwide intervention program. A few steps have already been taken to reduce the incidence and prevalence of obesity and, as a result, that of MetS, such as the introduction of the public health product tax (Népegészségügyi termékadó—NETA) in 2011. NETA is a single-phase consumption tax payable on the first domestic sale of various products containing sugar, salt, and caffeine. In addition to the many advantages of this type of tax, it should be emphasized that changes in consumption strongly vary by weight categories in the population. Overweight and obese individuals were twice as likely to change their consumption behaviors around unhealthy food and drink products than those who were underweight or of normal weight [53]. Although NETA has been included in the WHO good practice brief database [54], data of our 2015 and 2018 surveys on prevalence of overweight and obesity are not convincing in terms of its effectiveness [55] and the systematic Cochrane review concludes also that it is “uncertain whether taxing unprocessed sugar or sugar-added foods has an effect on reducing their consumption and preventing obesity or other adverse health outcomes” [56].

Despite the beneficial properties of NETA, it is clear from our results that its introduction failed to stop the increase in the frequency of metabolic syndrome in the Hungarian population. To date, no research has been conducted in Hungary that would have monitored long-term changes in metabolic syndrome and its components as a result of a specific intervention to reduce them. However, a number of studies have been conducted that have examined one or more elements of MetS and suggest targeted interventions to reduce it (such as diabetes and cardio-metabolic diseases). There is a definite need to develop a targeted prevention program at a national level with a focus on young adults.

To improve the effectiveness of preventive interventions against—among other things—cardio-metabolic disturbances and diseases:

- Primary health care should be reoriented toward public health services. As is described previously, general medical practice is basically limited to patient care and referral to specialized care in Hungary, and there is a lack of screening examinations ensuring early detection of high blood pressure, diabetes and lipid disorders, and lifestyle counselling aimed at preventing the development of chronic noncommunicable diseases [57];
- Nutritional counselling in primary care is rather sporadic; to insert it into the regular services of family practices would require changes in insurance regulation and the reimbursement system together with an increase in resources [58];
- Not only nutritional counselling, but other preventive services (as, for example, regular measurement of laboratory parameters indicative of the development and progression of cardio-metabolic diseases, assisting cancer screening, measuring anthropometric parameters relevant to the early detection of metabolic syndrome, etc.) are severely underutilized in Hungarian primary care [59,60];
- The patients’ willingness to utilize preventive services is also insufficient; our statement as we conclude on the basis of observation in a primary care model program we had conceptually developed and implemented in collaboration with other consortium members [57,61] that “The future of general practices lays in multidisciplinary teams in which health mediators recruited from the serviced communities can be valuable members, especially in deprived areas” [62] should be emphasized.

One of the advantages of the study is also a disadvantage, as the confounding effect of environmental factors cannot be ruled out in the case of samples from the same region during the comparative analysis. It is also a limitation that the two counties examined cannot be considered representative of the whole Hungarian population; however, these two counties are considered disadvantaged from a public health point of view, which is further aggravated by negative changes in the frequency of metabolic syndrome and its components.

It is also worth mentioning that the frequency of metabolic syndrome has been determined in many countries around the world, but its trend reversal is much less studied. These types of studies are needed to examine the effectiveness of targeted interventions that are supposed to reduce the frequency of MetS at the outcome level.

## 5. Conclusions

Our results clearly show that between 2006 and 2018, the frequency of metabolic syndrome increased in the Northeast Hungarian population. This change is mainly seen in women and is most severe in the 20–34-year-old age group in both sexes. Stopping this trend would definitely require a type of intervention with a focus on women and the 20–34-year-old age group.

## Figures and Tables

**Table 1:** Biochemical and physical parameters and the frequency of preventive medications used to estimate the prevalence of metabolic syndrome in the study populations.

**A**	**Sample of 2006**	**Sample of 2018**	***p*-Value**
	**Mean (95% CI)**	**Mean (95% CI)**	
Fasting glucose (mmol/L)	**4.6 (4.4–4.8)**	**5.2 (5.0–5.4)**	**<0.001**
Fasting TG (mmol/L)	1.7 (1.5–1.8)	1.6 (1.5–1.7)	0.721
HDL-C (mmol/L)	1.5 (1.4–1.5)	1.4 (1.3–1.4)	0.086
Waist circumference (cm)	94.5 (93.1–95.9)	95.9 (94.4–97.4)	0.279
Systolic blood pressure (mmHg)	126.6 (125.0–128.1)	126.8 (125.2–128.3)	0.352
Diastolic blood pressure (mmHg)	79.8 (78.9–80.6)	78.8 (77.8–79.7)	0.522
**B**	**Prevalence** **(95% CI)**	**Prevalence** **(95% CI)**	***p*-Value**
Prevalence of antihypertensive treatment (%)	29.8 (25.5–34.3)	29.7 (25.2–34.5)	0.986
Prevalence of antidiabetic treatment (%)	4.6 (2.9–7.0)	6.3 (4.1–9.1)	0.315
Prevalence of lipid-lowering therapy (%)	**15.9 (12.6–19.6)**	**7.1 (4.8–10.1)**	**<0.001**

Significant differences in mean or prevalence rates are highlighted in **bold**; 95% CI: 95% confidence interval.

**Table 2:** Change in the prevalence of metabolic syndrome and its components by sex in the population of Northeast Hungary between 2006 and 2018.

	Sample of 2006	Sample of 2018	*p*-Value	Male Samples of 2006 (*n* = 177)	Male Samples of 2018 (*n* = 147)	*p*-value	Female Samples of 2006 (*n* = 233)	Female Samples of 2018 (*n* = 220)	*p*-Value
MetS and Its Components	Prevalence % (95% CI)	Prevalence % (95% CI)		Prevalence % (95% CI)	Prevalence % (95% CI)		Prevalence % (95% CI)	Prevalence % (95% CI)	
Central obesity	70.7 (66.2–75.0)	75.5 (70.9–79.7)	0.137	62.7 (55.4–69.6)	64.6 (56.7–72.0)	0.722	76.8 (71.1–81.9)	82.7 (77.3–87.3)	0.119
Raised BP or treated hypertension	**45.6 (40.8–50.5)**	**57.0 (51.8–61.9)**	**0.002**	49.7 (42.4–57.0)	60.6 (52.5–68.2)	0.051	**42.5 (36.3–48.9)**	**54.6 (47.9–61.0)**	**0.010**
Raised FPG concentration or previously diagnosed diabetes mellitus	**13.2 (10.2–16.7)**	**24.8 (20.6–29.4)**	**<0.001**	**17.0 (12.0–23.0)**	**26.5 (19.9–34.1)**	**0.036**	**10.3 (6.9–14.7)**	**23.6 (18.4–29.6)**	**<0.001**
Raised TG level or treated lipid disorder	40.2 (35.6–45.1)	39.5 (34.6–44.6)	0.835	49.7 (42.4–57.0)	46.3 (38.3–54.3)	0.535	33.1 (27.3–39.3)	35.0 (28.9–41.5)	0.661
Reduced HDL-C level or treated lipid disorder	38.8 (34.2–43.6)	37.6 (32.8–42.6)	0.736	39.0 (32.0–46.3)	34.7 (27.4–42.6)	0.426	38.6 (32.6–45.0)	39.6 (33.3–46.1)	0.841
Metabolic syndrome	**34.9 (30.4–39.6)**	**42.2 (37.3–47.3)**	**0.035**	36.7 (29.9–44.0)	40.8 (33.1–48.9)	0.451	**33.5 (27.7–39.7)**	**43.2 (36.8–49.8)**	**0.034**

Significant differences in prevalence rates are highlighted in **bold**; 95% CI: 95% confidence interval; BP: blood pressure; FPG: fasting plasma glucose; TG: triglyceride; HDL-C: high-density lipoprotein cholesterol.

**Table 3:** Change in prevalence of metabolic syndrome and its components by age group in the Northeast Hungarian population from 2006 and 2018.

		Sample of 2006	Sample of 2018	*p*-Value
Age Groups	MetS and Its Components	Prevalence% (95% CI)	Prevalence% (95% CI)	
20–34	Central obesity	**44.4 (34.9–54.3)**	**62.2 (52.4–71.4)**	**0.012**
	Raised BP or treated hypertension	**13.1 (7.6–20.8)**	**36.7 (27.7–46.5)**	**<0.001**
	Raised FPG concentration or previously diagnosed diabetes mellitus	**1.0 (0.1–4.6)**	**7.1 (3.3–13.5)**	**0.029**
	Raised TG level or treated lipid disorder	21.2 (14.1–30.0)	30.6 (22.2–40.2)	0.132
	Reduced HDL-C level or treated lipid disorder	**21.2 (14.1–30.0)**	**36.7 (27.7–46.6)**	**0.016**
	Metabolic syndrome	**12.1 (6.8–19.6)**	**31.6 (23.1–41.3)**	**0.001**
35–49	Central obesity	70.4 (62.6–77.5)	76.8 (68.8–83.5)	0.239
	Raised BP or treated hypertension	**36.6 (29.0–44.8)**	**50.4 (41.7–59.1)**	**0.023**
	Raised FPG concentration or previously diagnosed diabetes mellitus	**6.3 (3.2–11.3)**	**20.8 (14.4–28.5)**	**<0.001**
	Raised TG level or treated lipid disorder	42.3 (34.4–50.5)	33.6 (25.8–42.2)	0.146
	Reduced HDL-C level or treated lipid disorder	42.3 (34.4–50.5)	36.0 (28.0–44.7)	0.297
	Metabolic syndrome	32.4 (25.1–40.4)	33.6 (25.8–42.2)	0.834
50–64	Central obesity	86.4 (80.6–90.9)	83.3 (76.6–88.7)	0.569
	Raised BP or treated hypertension	72.2 (65.1–78.5)	76.4 (69.0–82.8)	0.450
	Raised FPG concentration or previously diagnosed diabetes mellitus	**26.0 (19.9–33.0)**	**40.3 (32.5–48.4)**	**0.007**
	Raised TG level or treated lipid disorder	49.7 (42.2–57.2)	50.7 (42.6–58.8)	0.861
	Reduced HDL-C level or treated lipid disorder	46.2 (38.8–53.7)	39.6 (31.9–47.7)	0.242
	Metabolic syndrome	50.3 (42.8–57.8)	56.9 (48.8–64.8)	0.240

Significant differences in prevalence rates are highlighted in **bold**; 95% CI: 95% confidence interval; BP: blood pressure; FPG: fasting plasma glucose; TG: triglyceride; HDL-C: high-density lipoprotein cholesterol.

**Table 4:** The results of multivariate logistic regression models (adjusted by age and sex) to estimate the risk (OR—odds ratioand 95% CI—95% confidence interval) for metabolic syndrome and its components in the samples of 2018 compared to that of 2006 by age groups. The sample population of 2006 was used as reference.

	20–34		35–49		50–64	
MetS and Its Components	OR (95% CI)	*p*-Value	OR (95% CI)	*p*-Value	OR (95% CI)	*p*-Value
Central obesity	**1.12 (1.03–1.22)**	**0.008**	1.05 (0.97–1.14)	0.237	0.94 (0.86–1.04)	0.234
Raised BP or treated hypertension	**1.24 (1.12–1.38)**	**<0.001**	**1.08 (1.00–1.16)**	**0.047**	1.02 (0.95–1.10)	0.542
Raised FPG concentration or previously diagnosed diabetes mellitus	1.33 (0.98–1.80)	0.066	**1.21 (1.08–1.36)**	**0.001**	**1.10 (1.02–1.18)**	**0.009**
Raised TG level or treated lipid disorder	1.10 (0.99–1.21)	0.066	0.94 (0.88–1.01)	0.110	1.00 (0.94–1.07)	0.976
Reduced HDL-C level or treated lipid disorder	**1.124 (1.03–1.23)**	**0.012**	0.97 (0.90–1.04)	0.324	0.96 (0.90–1.02)	0.211
Metabolic syndrome	**1.21 (1.09–1.35)**	**0.001**	1.00 (0.93–1.08)	0.976	1.03 (0.97–1.10)	0.373

Significant differences in ORs are highlighted in **bold**; 95% CI: 95% confidence interval; BP: blood pressure; FPG: fasting plasma glucose; TG: triglyceride; high-density lipoprotein cholesterol: HDL-C.

**Table 5:** Change in the prevalence of untreated individuals for the four treatable components of metabolic syndrome (MetS) in study populations (A) and by sex (B).

**A**						
**MetS Components**	**Sample of 2006**		**Sample of 2018**		***p* -Value**	
	**Untreated in % (95% CI)**					
Raised BP or treated hypertension	**34.8 (28.2–41.8)**		**47.9 (41.1–54.6)**		**0.008**	
Raised FPG concentration or previously diagnosed diabetes mellitus	64.8 (51.6–76.5)		74.7 (65.1–82.8)		0.203	
Raised TG level or treated lipid disorder	**60.6 (53.0–67.8)**		**82.1 (75.2–87.7)**		**<0.001**	
Reduced HDL-C level or treated lipid disorder	**59.1 (51.4–66.5)**		**81.2 (74.0–87.0)**		**<0.001**	
**B**						
**MetS Components**	**Males**			**Females**		
	**Sample of 2006**	**Sample of 2018**	***p*-Value**	**Sample of 2006**	**Sample of 2018**	***p*-Value**
	**Untreated in % (95% CI)**			**Untreated in % (95% CI)**		
Raised BP or treated hypertension	43.2 (33.2–53.6)	55.1 (44.7–65.1)	0.114	**27.3 (19.2–36.6)**	**42.5 (33.9–51.4)**	**0.019**
Raised FPG concentration or previously diagnosed diabetes mellitus	80.0 (63.3–91.2)	74.4 (59.3–86.0)	0.582	**45.8 (27.3–65.3)**	**75.00 (62.1–85.2)**	**0.013**
Raised TG level or treated lipid disorder	**60.2 (49.8–70.0)**	**80.9 (70.4–88.8)**	**0.006**	**61.0 (49.9–71.4)**	**83.1 (73.6–90.2)**	**0.002**
Reduced HDL-C level or treated lipid disorder	**49.3 (37.7–60.9)**	**74.5 (61.4–84.9)**	**0.005**	**66.7 (56.5–75.8)**	**85.1 (76.5–91.4)**	**0.004**

Significant differences in prevalence rates are highlighted in **bold**; 95% CI: 95% confidence interval; BP: blood pressure; FPG: fasting plasma glucose; TG: triglyceride; HDL-C: high-density lipoprotein cholesterol.

## Data Availability

Data available on request due to privacy or ethical.

## Supplementary Materials

The following are available online at https://www.mdpi.com/2075-4426/11/1/52/s1, Supplementary Table S1. Anthropometric and demographic characteristics of the study populations, Supplementary Table S2. Biochemical, physical parameters and frequency of preventive medications used to estimate the prevalence of metabolic syndrome in the study populations by sex.
