# Supplementary material for: Changes in the Prevalence of Metabolic Syndrome and Its Components as Well as in Relevant Preventive Medication between 2006 and 2018 in the Northeast Hungarian Population
Source: J Pers Med. 2021 Jan 16;11(1):52. doi: 10.3390/jpm11010052 (PMC7829989; doi:10.3390/jpm11010052)
Supplement: Supplementary file 1 [file jpm-11-00052-s001.pdf]

**Table 1.** Anthropometric and demographic characteristics of the study populations.

| | | Sample of 2006 Prevalence % (n) | Sample of 2018 Prevalence % (n) | p-value |
|---|---|---|---|---|
| Sex | Male | 48.22 (177) | 44.58 (147) | 0.379 |
| | Female | 51.78 (233) | 55.42 (220) | |
| Age groups | 20-34 | 30.89 (99) | 24.69 (98) | 0.702 |
| | 35-49 | 31.56 (142) | 39.04 (125) | |
| | 50-64 | 37.56 (169) | 36.27 (144) | |
| | | Mean (95% CI) | Mean (95% CI) | p-value |
| Age (years) | | 44.51 (43.36-45.29) | 44.29 (42.99-45.59) | 0.890 |

95% CI: 95% confidence interval.

**Table 2.** Biochemical, physical parameters and frequency of preventive medications used to estimate the prevalence of metabolic syndrome in the study populations by sex.

| | **Males** | | | Females | | |
|---|---|---|---|---|---|---|
| **A** | **Sample of 2006** | **Sample of 2018** | **p-value** | Sample of 2006 | Sample of 2018 | p-value |
| | Mean (95% CI) | Mean (95% CI) | | Mean (95% CI) | Mean (95% CI) | |
| Fasting glucose (mmol/L) | **4.79 (4.50-5.08)** | **5.34 (4.98-5.70)** | **<0.001** | **4.47 (4.26-4.68)** | **5.15 (4.94-5.37)** | **<0.001** |
| Fasting TG (mmol/L) | 1.99 (1.67-2.31) | 1.85 (1.65-2.06) | 0.579 | 1.39 (1.28-1.50) | 1.39 (1.28-1.50) | 0.917 |
| HDL-C (mmol/L) | 1.35 (1.27-1.43) | 1.25 (1.19-1.30) | 0.176 | 1.53 (1.48-1.59) | 1.46 (1.41-1.52) | 0.150 |
| Waist circumference (cm) | 97.64 (95.66-99.63) | 98.52 (96.39-100.66) | 0.498 | 92.11 (90.18-94.05) | 94.15 (92.03-96.27) | 0.230 |
| Systolic blood pressure (mmHg) | 129.35 (127.26-131.44) | 129.56 (127.32-131.80) | 0.379 | 124.46 (122.29-126.63) | 124.91 (122.87-126.96) | 0.469 |

| | | | | | | |
|---|---|---|---|---|---|---|
| Diastolic blood pressure (mmHg) | 81.21 (79.87-82.56) | 80.14 (78.90-81.39) | 0.550 | 78.64 (77.49-79.79) | 77.81 (76.55-79.08) | 0.741 |
| B | | | | | | |
| Prevalence of antihypertensive treatment (%) | 28.25 (22.01-35.19) | 27.21 (20.51-34.80) | 0.836 | 30.90 (25.23-37.04) | 31.36 (25.51-37.71) | 0.915 |
| Prevalence of antidiabetic treatment (%) | 3.39 (1.43-6.85) | 6.80 (3.55-11.73) | 0.158 | 5.58 (3.16-9.09) | 5.91 (3.35-9.61) | 0.880 |
| Prevalence of lipid lowering therapy (%) | **19.77 (14.42-26.10)** | **8.84 (5.05-14.24)** | **0.006** | **12.88 (9.04-17.63)** | **5.91 (3.35-9.61)** | **0.011** |

Significant differences in mean or prevalence rates are highlighted in **bold**. 95% CI: 95% confidence interval.
